# Application of mild hypothermia successfully mitigates neural injury in a 3D in-vitro model of traumatic brain injury

**DOI:** 10.1371/journal.pone.0229520

**Published:** 2020-04-01

**Authors:** Mark T. Scimone, Harry C. Cramer, Paul Hopkins, Jonathan B. Estrada, Christian Franck

**Affiliations:** 1 School of Engineering, Brown University, Providence, RI, United States of America; 2 Center for Biomedical Engineering, Brown University, Providence, RI, United States of America; 3 Department of Mechanical Engineering, University of Michigan—Ann Arbor, Ann Arbor, MI, United States of America; 4 Mechanical Engineering, University of Wisconsin–Madison, Madison, WI, United States of America; University of Florida, UNITED STATES

## Abstract

Therapeutic hypothermia (TH) is an attractive target for mild traumatic brain injury (mTBI) treatment, yet significant gaps in our mechanistic understanding of TH, especially at the cellular level, remain and need to be addressed for significant forward progress to be made. Using a recently-established 3D in-vitro neural hydrogel model for mTBI we investigated the efficacy of TH after compressive impact injury and established critical treatment parameters including target cooling temperature, and time windows for application and maintenance of TH. Across four temperatures evaluated (31.5, 33, 35, and 37°C), 33°C was found to be most neuroprotective after 24 and 48 hours post-injury. Assessment of TH administration onset time and duration showed that TH should be administered within 4 hours post-injury and be maintained for at least 6 hours for achieving maximum viability. Cellular imaging showed TH reduced the percentage of cells positive for caspases 3/7 and increased the expression of calpastatin, an endogenous neuroprotectant. These findings provide significant new insight into the biological parameter space that renders TH effective in mitigating the deleterious effects of cellular mTBI and provides a quantitative foundation for the future development of animal and preclinical treatment protocols.

## Introduction

Despite the overwhelming amount of research and resources devoted, traumatic brain injury (TBI) continues to be a serious public health concern. In the US alone, there are an estimated 1.7 million new cases of TBI assessed in emergency rooms every year and incidence of sports-related concussions may approach 3.8 million annually.[1,2] The underlying cellular mechanisms of TBI are both complex and poorly understood, resulting in no widely agreed-upon treatments and unreliable diagnosis strategies.[3,4] As the list of diseases implicated with TBI–such as the neurodegenerative diseases Alzheimer’s and Parkinson’s[5–7]–continues to grow, clinicians and care givers search for a treatment that universally interacts with a wide range of biochemical pathways involved in TBI. Because changes in thermal energy can regulate nearly every biological function, therapeutic hypothermia (TH) has been suggested as an attractive potential treatment option. Currently, TH, sometimes referred to as targeted temperature management, is used in some clinical procedures, such as cardiopulmonary bypass surgery or circulatory arrest but not in the treatment of TBI.[8]

The documented benefits of TH in TBI studies include reduction of intracranial pressure, neuroexcitotoxicity, caspase enzyme activation, inflammation, cerebral edema, metabolic rate, and reactive oxygen species (ROS) generation.[9–13] Despite these potential advantages, there have been numerous studies that have resulted in neutral or unfavorable outcomes in clinical or preclinical TH treatment groups.[13–15] Complications of and from systemic, i.e., whole body, administration of hypothermia may include pneumonia, coagulopathy, and an increased risk of infection, among others.[9,13]

Patients with TBI often have comorbid injuries, complicating treatment and outcome. Complex symptoms and treatments make TH for TBI a difficult problem to study in clinical settings. Clinical methods have not been consistent between studies, resulting in differences in hypothermia administration duration, onset time, cooling rate, method of cooling; as well as differences in inclusion criteria.[16,17] The cellular ramifications can be substantial, as enzymatic processes in neural cell death pathways post-TBI are extremely time and temperature sensitive, which needs to be considered when designing clinical trials and applications protocols. Yet, to date detailed temporal and temperature information as well as guidelines for the potential use of TH are missing. Determination of some of the pertinent parameters for clinical treatment, which include optimal cooling temperature, critical time of cooling initiation, cooling rates and total duration of cooling administration, can be gleaned, at least initially, by studying the cellular response in physiologically relevant in-vitro models. Such systems offer high throughput, fine variable control and complete spatiotemporal access, and thus are an attractive approach for initially establishing a successful TH parameter space to subsequently inform animal and preclinical studies of TH. Furthermore, because of the confounding and comorbid factors accompanying moderate and severe TBI, accurate conclusions are likely easier to be deduced from TH’s effects on mild TBI (mTBI), which we aim to simulate here.

**Fig 1:**
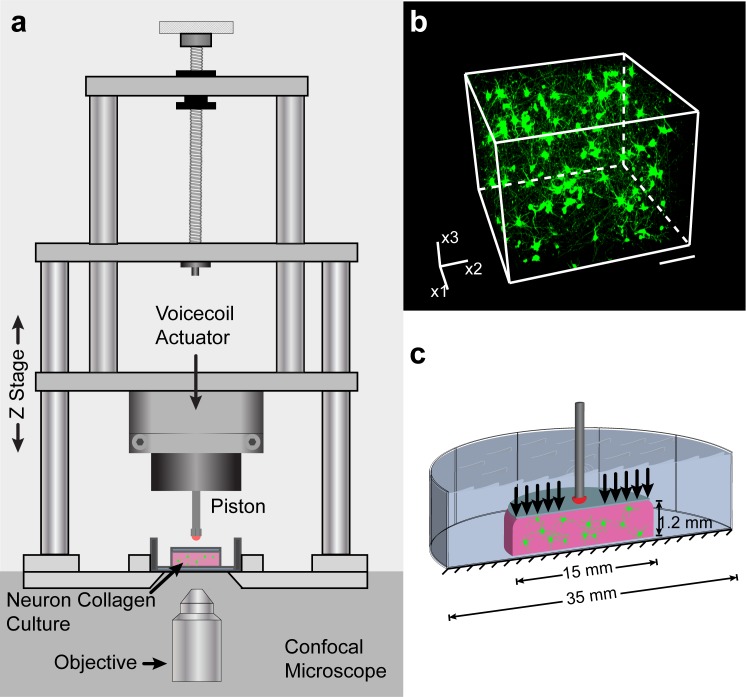
Experimental set up. (**a**) Uniaxial compressive impact is generated by a linear voicecoil actuator. The cellular injury device (characterized in Bar-Kochba et al., 2016 & used in Scimone et al., 2018) is mounted on a confocal microscope stage in a temperature-controlled chamber.[20,21] (**b**) 3D confocal micrograph showing mature cellular neural network architecture. (**c**) Schematic illustration of the uniaxial compressive impact strains imparted onto the neural cells.

To accurately study TBI at the cellular level, the field requires 3D, physiologically-relevant in-vitro research[18,19]. Using a 3D, in-vitro neural injury model of mTBI previously described in Bar-Kochba et al. (*Sci Rep*, 2016) and in Scimone et al. (*Nat Prot*, 2018)[20,21] (Fig 1), we studied the efficacy of therapeutic hypothermia treatment after compressive injury, initially focusing on the optimal target temperature and the role of TH administration onset time and duration. Using live cell confocal microscopy, we found that the optimal neuroprotective temperature is 33°C and that TH should be administered within 4 hours post-injury and for at least 6 hours to achieve maximum viability benefits. Further examination investigated the effect of TH on the activity of several well-known apoptotic pathways involved in cellular death after mTBI, namely: caspase 3 and 7 activation, reactive oxygen species generation, and calpastatin expression. Through a combination of live- and fixed-cell fluorescent confocal imaging, we found that TH significantly decreased caspase activation and increased expression of calpastatin, which is a known neuroprotectant.[22] We assert that these cell-derived measurements provide valuable insight and guidance for informing further in-vivo and preclinical work.

## Materials and methods

### Study design

#### Sample preparation and maintenance

All experiments involving animals were approved by the IACUC of Brown University and performed in accordance with their guidelines set forth. All sample preparation and tissue culture steps are based on our previously demonstrated 3D cellular mTBI model.[20,21] This model contains neurons, astrocytes, and neural progenitors.

For cellular isolation and culture, primary cortical neural cells were harvested from postnatal Sprague-Dawley rats (p0-p1). The isolated cells were then encapsulated in collagen hydrogels (type 1, final concentration of 2 mg/mL) molded in custom-made Delrin molds. Cylindrical gels were placed in individual wells and submerged in culture media (consisting of neurobasal–A medium + 2% 50x B-27 supplement +1% mixture of Penicillin Streptomycin, L-glutamine, and fetal bovine serum; all reagents are from Life Technologies, Grand Island, NY) pre-warmed to 37°C. The samples were allowed a seven-day growth period in a sterile incubator (37°C, 5% CO_2_) to undergo synaptogenesis and create networks of neurons, astrocytes, and neural progenitors. See Fig 1B for a 3D volume confocal micrograph showing a typical network of neural cells. During this time, media was changed one day after harvest, and then every two to three days thereafter. See Fig 1C for a schematic of the hydrogel with neural cells in it.

#### Method of injury

Neural cells encapsulated in the collagen hydrogels were injured via uniaxial compression (max nominal strain = 0.26). Strain pulses were chosen based on reported values for peak strain and strain rate reconstructed from literature studies on concussions[23,24]. Injury strains were chosen to be representative of the tissue strains experienced in mTBI. A linear voice-coil actuator was used to enable precise control over the nominally applied strain and strain rate following the same load-unload profile as in Bar-Kochba et. al 2018 with a peak strain rate of 89.1 s^-1^. A detailed description on the resolution of locally experienced 3D neural strains and strain fields indicative of mTBI, as well as detailed experimental validation of the impact platform, can be found in our previous work[20]. All impacts were performed at 7 days in-vitro (DIV), following the initial network growth and formation period. To ensure that cells experienced uniform and well-characterized 3D impact strains we followed the same experimental protocols as previously documented[20,21].

### Hypothermia regulation and Rate of cooling

Immediately following impact or sham impact, all samples were transferred to an incubator set to the target temperature and submerged in pre-warmed media. Three temperature groups were chosen: 35, 33, and 31.5°C. Rate of cooling was estimated via the classical heat diffusion solution (Brown University Thesis, Mark Scimone, 2019), as well as measured via a thermocouple placed at the center of the sample.[25] According to the diffusion solution we expect that 95% of the sample reaches the target temperature within less than a minute for all conditions, which was in agreement with our experimental measurements.

#### Confocal microscopy

All confocal micrographs were obtained using a Nikon A1R confocal system mounted on a Ti-Eclipse inverted optical microscope. The system was controlled by NIS-Elements Nikon software (Nikon, Tokyo, Japan). Cell population viability was assessed using the fluorescent stains calcein AM and ethidium homodimer-1. Calcein AM (ThermoFisher Scientific) readily infiltrates cell membranes and fluoresces when cleaved by intracellular esterases during excitation via an argon ion laser (488 nm). In this way, calcein AM was used to visualize live cells. Ethidium homodimer-1 (EthD-1; ThermoFisher Scientific) is cell-impermeant, but fluoresces when bound to nuclear DNA and excited by a HeNe diode laser (561 nm), and was used to stain dead cells. Samples were stained with both dyes for 1.5 hours before imaging (16 μM of calcein AM and 12 μM of EthD-1 in Hibernate-A/B-27). The large sample geometry and cell seeding density required a higher concentration than recommended by the manufacturer. Control experiments confirmed that the addition of the dyes by themselves in the absence of applied strain did not produce any loss in viability. Confocal micrographs were recorded and collected at either 24 or 48 hours post impact, depending on the experiment. 3D confocal micrographs of 512 x 512 x 200 voxels (635 × 635 × 248 μm^3^) were collected using a 20X / 0.45 objective (S Plan Fluor ELWD Ph1 ADM, Nikon). Z-step size was 1.24 μm, as is the native μm-to-pixel ratio of the objective. All maximum intensity projections shown in figures are through-thickness; i.e. all slices of the confocal volume are projected. The confocal microscope system is contained in a temperature-controlled chamber, which was set to match the target temperature of each sample group.

#### Population health assessment

Two channels of fluorescence emission signal, one corresponding to live cells and one to dead cell nuclei were recorded simultaneously via confocal microscopy. Previously developed in-house image processing algorithms account for and reduce signal noise and correct for bidirectional pixel mismatch[20]. Cell population health was determined by manual counting in FLNeuronTool[26]; all counts were verified by at least two lab members individually.

#### Caspase 3& 7 and reactive oxygen species assessment

To measure live cells positive for caspase 3 or 7, CellEvent^™^ green ReadyProbes^™^ (ThermoFisher Scientific) was used (2 drops/mL). To measure live cells positive for ROS, CellROX^™^ green (ThermoFisher Scientific) was used (4 μM). In both cases, cell populations were counterstained with Hoechst 34580 (ThermoFisher Scientific) to show the nuclei and EthD-1 to show dead cell nuclei (6 μM and 12 μM, respectively). In this way, cells were considered positive for the marker-of-interest if the fluorescence signal colocalized with the Hoechst stain signal, but not the EthD-1 stain signal. The colocalization assessment was automated using image processing techniques in MATLAB, detailed below. Stuarosporine, a compound known to cause apoptosis, was administered to cells for the positive caspase control group at 0.5 μM for 16 hours prior to fluorescent staining. Menadione, a compound known to increase cellular ROS generation, was administered to cells for the positive ROS control group at 50 μM for 1 hour prior to fluorescent staining.

### Caspase 3 & 7 and ROS image analysis

A custom Matlab code was developed to identify the number of cells positive for a fluorescent marker of interest. Here the Caspase and ROS assay image datasets were analyzed identically. Briefly, 1) Each channel was binarized based on its histogram of intensities. 2) All images were median filtered and de-speckled. 3) A bounding box was found for each semi-spherical object. 4) In each channel, the bounding box objects were checked for overlap with bounding boxes in the other two channels. Overlapping signal indicates colocalization. 5) Cells were considered positive if their caspase-3/7 or ROS signal colocalized with the Hoechst signal, but not the EthD-1 signal.

#### Cryosectioning

Cryosectioning and immunostaining were performed based on adapted protocols from Dingle et al., 2015 [27]. Collagen/neural cell cultures were fixed in 4% v/v paraformaldehyde and 8% w/v sucrose in phosphate-buffered saline (PBS) for 1hr at 4°C, washed with PBS thrice, and prepped for cryosectioning by embedding in optimal cutting temperature (OCT; Fisher Scientific) compound and freezing on dry ice. The samples were then cut into 20 μm slices using a Leica CM3050 S cryostat or an Avantik QS12 cryostat at -15°C. Sections were placed on SuperFrost Plus slides (Fisher Scientific) and kept at -20°C until immunofluorescence staining.

#### Immunofluorescence staining and imaging

Collagen/neural cell sections were brought to room temperature and fixed again with 4% v/v paraformaldehyde and 8% w/v sucrose in PBS for 5 minutes. The sections were washed twice with PBS, permeabilized with 0.1% Triton X-100 (TX) in PBS (PBT) for 15 minutes, and washed twice more with PBS. The samples were incubated overnight with primary antibody (anti-calpastatin, 1:50; ThermoFisher, Grand Island, NY, 1F7E3D10) in a dark, humidified chamber at 4°C. Following incubation with primary antibody, the samples were washed twice with PBT for 30 minutes each. Samples were then washed once with 1X PBS. Next, they were incubated with the secondary antibody (Alexa Fluor 555 goat anti mouse, 1:200; ThermoFisher Scientific, cat no. A28180) in blocking solution for 1 hour at room temperature while covered in aluminum foil to block light. After secondary staining, they were washed twice with PBT in the dark for 10 minutes each. They were then counterstained with 4′,6′-diamidino-2-phenylindole (DAPI; 300 mM) in PBT for 5 minutes at room temperature. After washing once more with 1X PBS for 5 minutes, the samples were mounted with Fluoromount (ThermoFisher Scientific) and left to cure overnight at room temperature, protected from light. Final mounted slides were sealed with nail polish prior to imaging so as to not have the coverslip move while imaging on the confocal microscope. 3D confocal micrographs of 1024 x 1024 x 40 voxels (159 × 159 × 20 μm^3^) were collected using a 40X / NA 1.15 objective (CFI Apo LWD Lambda S 40XC WI, Nikon).

Laser power, offset, and detector gain parameters were chosen based on control samples and were kept identical for all samples across experimental groups. This strict control of imaging parameters is critical for accurate comparisons of signal in fluorescent confocal microscopy.

### Immunofluorescence image analysis

Values for calpastatin expression are reported in AU, or arbitrary units. A custom MATLAB code was used to semi-automatically process the data. The image analysis routine was as follows. 1) Find the outline of the cell via a level-set segmentation of the brightfield channel. 2) Mask the cell in 3D using the brightfield boundary and DAPI signal in z to bound the mask. 3) Median filter the image. Lastly, 4) calculate the signal intensity within the cell boundary, requiring a minimum signal to noise of two standard deviations above the noise floor (normalized by the maximum bit-depth intensity).

### Statistical analysis

All statistical analysis was performed using SigmaPlot (Systat Software Inc., San Jose, CA). Analysis of variance (ANOVA) on ranks test was used to analyze differences between groups. Where data sets passed the test for normality, a Tukey test was used to calculate p-values. Where data sets did not pass the test for normality (sets shown in box and whisker plots that include outliers which were deemed not appropriate to remove), Dunn’s test was used to calculate p-values. Data shown in Fig 4 indicates all pairwise comparisons via Tukey test. All statistical comparisons were performed on a between-population basis.

## Results

**Fig 2:**
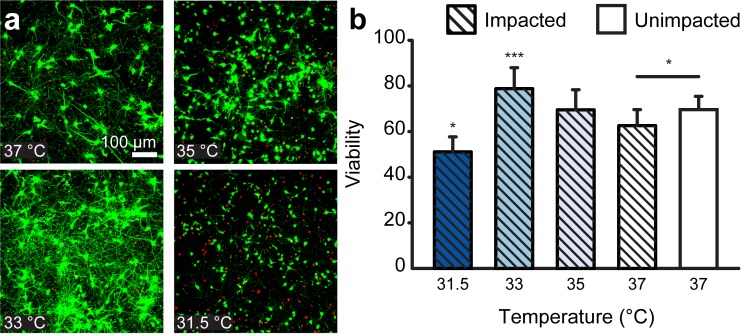
Population viability at hypothermic temperatures at 24 hours post-injury. (**a**) Representative maximum-intensity-projected confocal micrographs of cell populations after post-injury hypothermia treatment. Green cells are stained with calcein AM, indicating a live cell. Red staining indicated a dead cell nuclei stained with ethidium homodimer-1. (**b**) Effect of temperature on cell population viability after compressive injury. *, p < 0.05; ***, p < 0.001.

Neural injury was produced via our previously established uniaxial compression impact model (Fig 1A),[20,21] where the impact pulse was informed by literature values from head impacts,[23,24] yielding average peak strains of 26% at an average peak strain rate of 89.1 s^-1^. Samples were impacted after 7 DIV and subsequently placed in an incubator at each of the selected hypo- and normothermic temperatures. Numerical estimates of temperature measurements during the cooling and re-warming showed that average cooling and re-warming rates achieved the desired tissue temperature within less than a minute without any noticeable effect on viability. In the experiment, to first identify the ideal TH target temperature, population viability was assessed 24 hours post-injury using live-cell confocal microscopy. Specifically, population viability is assessed through a standard calcein AM, Ethidium homodimer-1cellular viability assay, where calcein AM (green) represents healthy intracellular esterase activity, and ethidium homodimer (red) staining indicates a loss of membrane integrity and cellular homeostasis. The resulting mean viabilities of impacted samples were 65.2, 69.7, 78.9, and 51.3 percent for 37, 35, 33, and 31.5°C, respectively (Fig 1B). The 33°C group had significantly higher viability (p<0.001), and the 31.5°C had significantly lower viability compared with the impacted, 37°C group (p<0.05). Fig 2A shows representative maximum-intensity-projected confocal micrographs for each group. It is important to note that the observed baseline viability of ~73% in healthy, uninjured controls is reflective of total cellular populations seeded from dissected cortical tissue. We have previously shown that the culture platform utilized here is not representative of oligodendrocyte or microglial populations present during cortical tissue isolation[21]. Populations of oligodendrocytes and microglia have been reported to be present at 17% and 9% respectively[28]. In line with these observations the authors affirm that the reported culture platform represents a healthy co-culture of neurons, astrocytes and neural progenitor cells, as previously demonstrated[21]. See S1 Fig for representative maximum intensity projected confocal micrographs of unimpacted controls at each experimental temperature.

**Fig 3:**
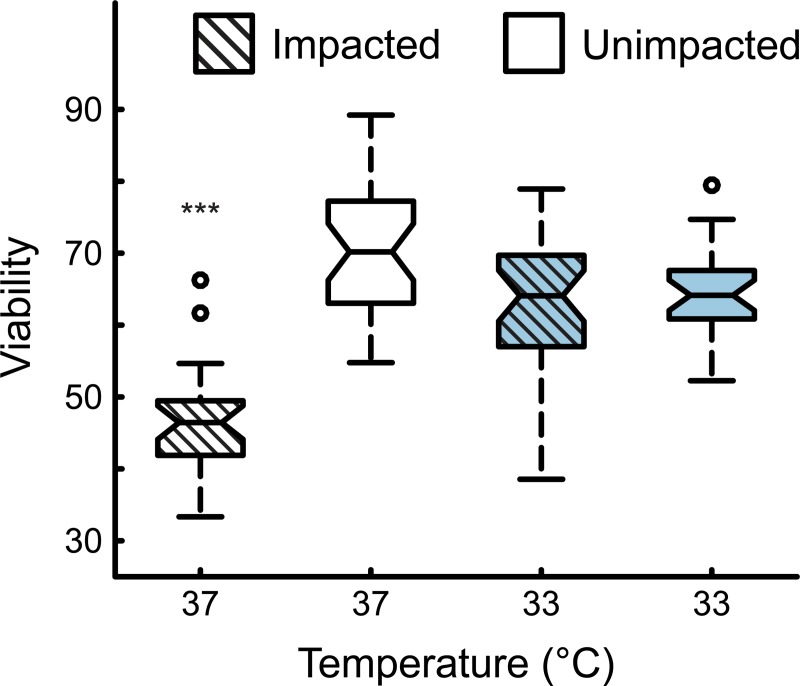
Longitudinal experiment at 48 hours post-injury. Viability of cell populations following 24 hours of TH treatment at 33°C and subsequent recovery for an additional 24 hours at 37°C, assessed 48 hours after injury. ***, p < 0.001.

After finding a statistically significant higher population viability after injury with the 33°C hypothermic treatment for 24 hours, a longitudinal study was conducted to check whether potential neuroprotective effects were transient or permanent (Fig 3). To this end, samples were exposed to hypothermia at 33°C for 24 hours immediately post impact. After 24 hours, samples were returned to 37°C for 24 more hours at normothermia. Live cell confocal imaging commenced after 48 total hours. The results after 48 hours showed that the injured, normothermia 37°C group had a viability of 46.4%. The injured, hypothermia-treated group at 33°C for 24 hours had a viability of 64.1%. The uninjured, hypothermia- (33°C for 24 hours, 37°C for 24 hours) treated control group had a 64.2% viability. The uninjured, normothermia (48 hours at 37°C) control group had a 70.2% viability. The injured normothermia (48 hours at 37°C) group was statistically different from the other three groups. The injured and treated 33°C, and two uninjured controls were statistically indistinguishable (Fig 3), showing that immediate cooling to 33°C for 24 hours had a significant neuroprotective effect against cellular mTBI.

**Fig 4:**
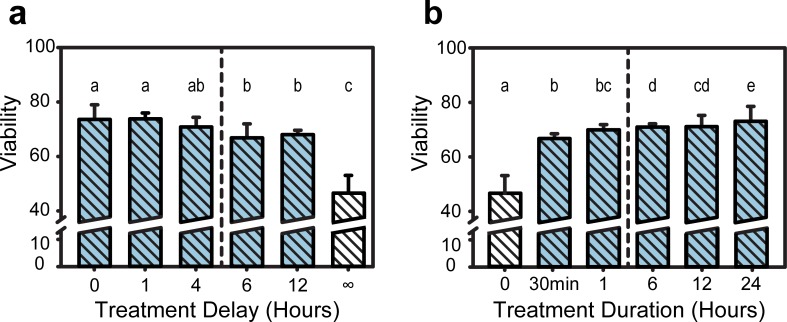
Population viability at varying treatment durations and initiation delay times. (**a**) The effect of TH treatment delay on cell population viability following compressive injury. Groups with matching letters are not statistically significant from each other. Differing letters indicate p < 0.05. Populations to the left of the dotted line are significantly more viable than those to the right (p < 0.05). (**b**) The effect of TH treatment duration following compressive injury on cell population viability. Groups with matching letters are not statistically significant from each other. Differing letters indicate p < 0.05. Populations to the right of the dotted line are significantly more viable than those to the left (p < 0.05).

To provide relevant TH administration time parameters, a delay and abbreviation study was conducted. A significant drop in viability occurred in injured samples if TH administration was delayed more than 4 hours (p<0.05, see Fig 4A). The mean viability percentage values were 73.6, 73.8, 71.1, 67.5, and 68.5 for 0, 1, 4, 6, and 12 hours of delay, respectively. In truncated TH administration time experiments, population viability significantly dropped if TH was administered for under 6 hours (p<0.05, see Fig 4B). The mean viability percentages were 67.4, 70.0, 71.6, 71.7, and 73.6 for 30 minutes, 1, 6, 12, and 24 hours of total TH treatment, respectively. It is important to note that the normothermic injured group, shown as ∞ hours and 0 hours of treatment delay and duration in Fig 4A and Fig 4B, respectively, had significantly lower viability than all other groups.

**Fig 5:**
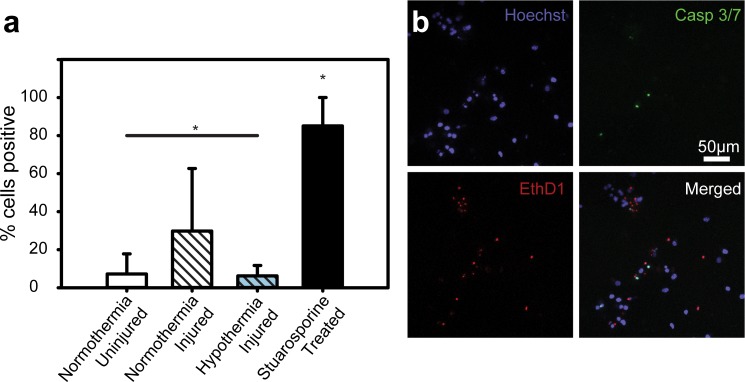
Effect of TH on caspase 3 and 7 activation. (**a**) Percentage of cells positive for activated caspase 3 or activated caspase 7. *, p < 0.05. (**b**) Representative maximum-intensity-projected confocal micrographs of cell populations stained with Hoechst 34580, CellEvent Green, and ethidium homodimer-1. Colocalization of CellEvent Green and Hoechst, with no ethidium homodimer-1 signal indicates a positive cell. 1.

To better understand how TH administration changed activity of executioner caspases, cells were assayed for activated caspase 3 or caspase 7 using a fluorescent reporter and live cell confocal microscopy. A significantly smaller percentage of cells were positive for caspase 3 and 7 in the injured TH-treated group (6.2%) compared to the normothermia injured (29.8%) counterpart (p<0.05, see Fig 5A). Furthermore, the injured TH-treated group percentage of positive cells did not differ statistically from the uninjured normothermia group (7.2%), which shows that the application of mild hypothermia provides a significant neuroprotective effect by maintaining caspase 3 and 7 expression levels similar to sham controls. All groups had a significantly lower percentage of cells positive when compared to the stuarosporine-treated positive controls (85.1%). See Fig 5B for representative maximum-intensity-projected confocal micrographs of each stain condition of the executioner caspase activation assay.

**Fig 6:**
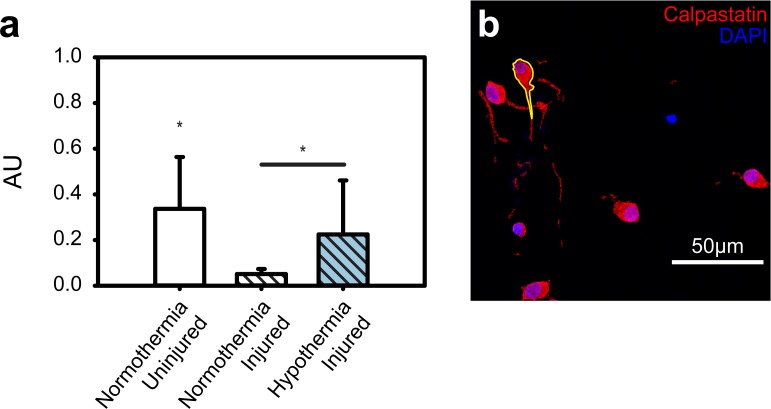
Effect of TH on calpastatin expression after compressive injury. (**a**) Calpastatin expression reported as arbitrary units (AU), calculated by normalizing the mean pixel intensity in the cell by the maximum value based on the bit depth. (**b**) Representative maximum-intensity-projected confocal micrograph of cells stained for calpastatin and counterstained with DAPI.

Whereas executioner caspases induce cell death, calpastatin protects against proteolysis by inhibiting calpain cytoskeletal proteolysis. To assay the neuroprotective results of TH administration on calpastatin, immunofluorescent staining and fixed cell confocal microscopy was used. The uninjured control had the highest expression (0.34 arbitrary units; AU), followed by the injured TH-treated group (0.22 AU), followed lastly by the injured untreated group (0.05 AU; see Fig 6A). All groups were statistically distinct (p<0.05). See Fig 6B for representative maximum-intensity-projected confocal micrographs of each stain condition; an example cell outline is shown in yellow.

The reactive oxygen species assay yielded no statistical difference between groups except for the menadione-treated positive control, which was statistically distinct (see S2A Fig). S2B Fig shows representative maximum-intensity-projected confocal micrographs of each stain condition of the reactive oxygen species assay.

## Discussion

Using our recently established 3D cellular mTBI model,[20,21] we conducted an extensive parameter sweep across a range of previously reported hypothermic temperatures and TH administration and initiation times to determine optimal cooling parameters for maintaining cellular viability post insult. As much of the literature on TH has provided conflicted accounts on its efficacy, this study aimed to provide clarification on a cellular and molecular basis for detailing under which conditions TH can be successful. We found that mild hypothermia at 33°C, administered immediately following impact for 24 hours, had the most significant neuroprotective effect by maintaining viability levels close to the unimpaired sham controls within our 3D neural cell-collagen cultures. We further confirmed that this neuroprotective effect remained unaltered within the time scales of our experiments (48 hours), which is qualitatively consistent with beneficial outcomes in recent applications of TH in animals and humans.[29,30]

We also determined that, once TH is administered, it should be continued for at least 6–12 hours to maintain maximum cell viability. Nevertheless, even shorter duration cooling, as long as administered within the first 4 hours, helped to maintain relatively high viability levels (Fig 4), which might help establish valuable guidelines for future animal and pre-clinical follow on work. The significance of the 4-hour time mark is in large part driven by the upregulation kinetics of a variety of proteins and enzymes known to cause apoptosis in cellular TBI[31] of particular note are the two executioner caspases—caspase 3 and 7. Both caspases perform many redundant roles in apoptosis, though caspase-3 is considered to act on more substrates.[32–34] For example, caspase-3 has been shown to cleave cytoskeletal components[35,36] and activate DNA fragmentation pathways.[37] There has been some evidence that temperature reduction might lessen the activation of these executioner caspases,[38–40] which is consistent with our results (Fig 5). Our data shows significantly reduced levels of both caspase 3 and 7 (Fig 5) as long as TH is administered within the first 4 hours, leading to a clear benefit in maintaining high cell population viability. Similar outcomes, consistent with our data, were reported by two recent randomized controlled clinical trials, showing poor outcomes in hypothermia treated groups that were unable to receive appropriate cooling temperature within the first 4 hours.[15,41] This and future work might thus underscore the need to begin treatment as soon as practically possible, with perhaps little benefit beyond a certain time point, e.g., after 12 hours. Unfortunately, at present, the road to admitting a patient with a TBI to TH clinical trials is plagued with delays, which can be associated with co-morbidity treatments, travel time, and obtaining consent, among others.

Therapeutic Hypothermia also affects the activity of innate defenses against calpains. Calpains are calcium dependent proteolytic enzymes that have also been shown to cleave cytoskeletal elements, such as spectrin and fodrin,[35,36,42–44] sometimes leading to apoptosis or necrosis. Of importance to the brain, calpains have been shown to cleave voltage-gated sodium channels[45]. Instead of quantifying calpain proteolysis directly, we investigated the cells innate ability to inhibit calpains via calpastatin. Calpastatin is an endogenous calpain inhibitor, which has been shown to have temperature-dependent activity. Reduced temperature has also been shown to decrease calpain-mediated proteolysis[46]. Our results here are consistent with those findings, indicating that a cell’s innate proteolysis inhibition can be strengthened with a mild reduction in temperature. Although our cellular mTBI model lacks much of the sophistication and the complexity of the true in-vivo environment, we believe it nevertheless provides a fundamental physical basis for helping inform the parameter space for further investigations of TH as a potential TBI treatment. The absence of significant amounts of glial cells, microvasculature and/or an innate immune response are all limiting factors of our current in-vitro system, and it will be interesting for future studies to investigate the attenuation of the TH results presented here within the context of multicellular, organoid-level interactions or an in-vivo model.

It is also important to point out that all of our cooling and re-warming rates were rapid, i.e., target temperatures were usually reached within about 15 seconds, a scenario that is unlikely implementable for the brain as a whole, especially in a clinical setting or for patients with co-morbidities. As there continues to be significant debate in the literature on the effect of cooling and re-warming rates on the outcome of TH, we hope to address these and other challenges in future studies. Likewise, the 31.5°C impacted group is noteworthy as it fared significantly worse than every other group. Though only 1.5°C lower than the neuroprotective zone, the viability was far lower. Indeed, many other studies have shown evidence of deleterious effects when cooling below 32°C[30,47,48], consistent with our findings here.

## Conclusion

The study presented here provides significant evidence that mild therapeutic hypothermia (33°C) administration has a neuroprotective effect on cortical neural cells subjected to compressive impact. Impact pulses were designed based on reconstructions from literature studies on concussion and thus were chosen to emulate mild TBI. The neuroprotective effect continued to persist 48 hours after injury at levels close to the unimpacted control samples. Furthermore, our data shows that TH should be administered within 4 hours of injury and for at least 6 hours to provide maximal cellular viability outcomes. Examination of the injury-associated, characteristic pathways of TBI, applications of mild TH significantly reduced caspase 3 and 7 activation and increased calpastatin expression after injury. In sum, this study provides significant fundamental insight into the role and effect of mild hypothermia on the survivability of neural cells, and shows that if the temperature, administration time and duration are carefully controlled, mild hypothermia can be a potent neuroprotective therapy against the deleterious effects of mTBI, at least at a cellular level. As such, this study may provide a foundation to inform future animal, pre-clinical and clinical studies to deliver a promising mTBI therapeutic that is both simple and effective.

## Limitations and future work

A significant strength of our model, as in many in-vitro models, is its simplicity and full access for spatiotemporal imaging, providing it with an ideal vantage point for the type of parameter space screening presented here. However, at the same time, in-vitro systems cannot address all the bodily responses involved in TBI. By design, our neural in-vitro system focuses on the behavior of neurons, astrocytes, and neural progenitors in our 3D cultures[21]. The native brain contains many more cell types, as well as cells associated with its vasculature. A common consequence of TBI is damage to, or temporary permeability in the blood brain barrier (BBB)[49]. In an uninjured state, the blood brain barrier carefully limits what can pass from the vasculature into the brain tissue. Damage to this can exacerbate the injury in TBI and is not replicated or captured by our system because it lacks a BBB simulant. For this reason, the system is likely not specialized enough to capture TH’s effect on ROS production after injury. There is evidence to support that a decrease in temperature downregulates ROS production, but most is related to mitochondrial function in muscle[50,51]. More research should be done on ROS production after TBI in systems that incorporate a BBB.

By providing new quantitative details on the efficacy of hypothermia in negating the deleterious effects associated with TBI at a cellular level, future studies will allow for the evaluation of the presented parameters within the context of repeat or multiple impacts. Future studies will also be able to examine the current hypothermic parameters within models of TBI of increased anatomical complexity and fidelity, such as organoids, organotypic slices, and animal models. Specifically, we plan to adapt the TBI system for organoids, such that soluble biomarkers implicated in TBI (*e*.*g*. UCH-L1, GFAP, and NF-l) can be detected and quantified.

## Supporting information

S1 Fig Control population viability at hypothermic temperatures at 24 hours. Representative maximum-intensity-projected confocal micrographs of control cell populations after hypothermia treatment. Green cells are stained with calcein AM, indicating a live cell. Red staining indicated a dead cell nuclei stained with ethidium homodimer-1.
(EPS)

S2 Fig Effect of TH on reactive oxygen species generation. (A) Percentage of cells positive for reactive oxygen species. *, p < 0.05. (B) Representative maximum-intensity-projected confocal micrographs of cell populations stained with Hoechst 34580, CellROX green, and ethidium homodimer-1. Colocalization of CellROX green and Hoechst, with no ethidium homodimer-1 signal indicates a positive cell.
(EPS)
